# The 90% effective dose (ED90) of remimazolam for inhibiting responses to the insertion of a duodenoscope during ERCP

**DOI:** 10.1186/s12871-024-02554-1

**Published:** 2024-05-14

**Authors:** Yan Guo, Shu-An Dong, Jia Shi, Hui-Rong Chen, Sha-Sha Liu, Li-Li Wu, Jing-Hua Wang, Li Zhang, Huan-Xi Li, Jian-Bo Yu

**Affiliations:** 1 Department of Anesthesiology, Heji Hospital Affiliated to Changzhi Medical College, Changzhi Medical College, Changzhi, Shanxi China; 2 Department of Anesthesiology and Critical Care Medicine, Tianjin Nankai Hospital, Tianjin Medical University, Tianjin, 300100 China; 3 Institute of Integrative Medicine for Acute Abdominal Diseases, Tianjin, China; 4 Tianjin Key Laboratory of Acute Abdomen Disease Associated Organ Injury and ITCWM Repair, Tianjin, China; 5 Department of Epidemiology, Tianjin Neurological Institute, Department of Neurology, Tianjin Medical University General Hospital, Tianjin, China; 6 Department of Hepatobiliary Surgery, Tianjin Nankai Hospital, Tianjin Medical University, Tianjin, China

**Keywords:** 90% effective dose, Remimazolam, Alfentanil, ERCP

## Abstract

**Background:**

Compared to midazolam, remimazolam has a faster onset and offset of hypnotic effect, as well as cardiorespiratory stability, this study aims to determine the 90% effective dose (ED90) of remimazolam to inhibit responses to insertion of a duodenoscope during endoscopic retrograde cholangiopancreatography (ERCP).

**Methods:**

A dose-response study was carried out undergoing ERCP who received remimazolam-alfentanil anesthesia using 10 µg/kg of alfentanil between September 2021 and November 2021. The initial dose of remimazolam was 0.2 mg/kg. The dose was then decided based on the responses of earlier patients by exploiting the sequential ascend and descend according to a 9: 1 biased coin design. Upon failure, the dose of remimazolam was increased by 0.025 mg/kg in the next patient. When the insertion was successful, the succeeding patient was randomized to an identical dose or a dose that was lower by 0.025 mg/kg.The ED90 of remimazolam for inhibiting responses to the insertion of a duodenoscope during ERCP was calculated. Adverse events and complications of remimazolam were recorded.

**Results:**

A total of 55 elderly patients (age > 65) were included in the study. 45 successfully anesthetized patients, and 10 unsuccessfully. The ED90 of remimazolam was 0.300 mg/kg (95% CI = 0.287–0.320). ED95 was 0.315 (95% CI = 0.312–0.323) and ED99 was 0.323 (95% CI = 0.323–0.325). Among the patients, 9 patients developed hypotension, 2 patients developed bradycardia and 1 patient developed tachycardia, and hypoxia occurred in 2 patients.

**Conclusions:**

A loading dose of 0.300 mg / kg of remimazolam for elderly patients undergoing ERCP can safely, effectively, and quickly induce patients to fall asleep and inhibit responses to the insertion of a duodenoscope.

**Trial registration:**

The study protocol was registered at the website ClinicalTrials.gov on 22/09/2021(NCT05053763).

## Introduction

A fundamental technique for diagnosing and managing pancreaticobiliary conditions is endoscopic retrograde cholangiopancreatography (ERCP) [1]. About one-third to half of the patients receiving ERCP under procedural sedation report feeling discomfort and pain [2]. To reduce laryngeal reflexes and avoid undesired body movements, nausea, coughing, and gagging, adequate sedation depth is required. A popular inducer administered during ERCP is propofol since it possesses sedation inhibitory properties [3, 4]. Unwanted responses cannot be fully inhibited by the anesthetic effects of propofol alone, while an overdose of propofol can impair the cardiorespiratory system unintentionally. To diminish the propofol demand and hemodynamic alterations, strong and short-acting opioids (e.g., alfentanil or fentanyl) are usually co-administered with propofol during ERCP. However, administering such opioids might increase the occurrence of hypoxia.

As a new short-acting benzodiazepine [5–10], remimazolam provides effective procedural sedation with superior success rates and recovery profile compared with midazolam [9]. However, comparing remimazolam to propofol for general anesthesia, the time to loss of consciousness and extubation were significantly longer in the remimazolam group [8]. Although the overall incidence of adverse drug reactions was similar in the remimazolam and propofol groups, fewer patients experienced hypotension in remimazolam group [8].

We conducted a randomized trial evaluated the efficacy and safety profiles of the remimazolam combined with alfentanil for sedation during ERCP procedures [11]. As a pilot study of this clinical trial, we determined the ED90 of remimazolam among elderly patients receiving ERCP.

## Methods

This study was approved by the Tianjin Nankai Hospital’s Clinical Trial Ethics Committee (NKYY_YXKT_IRB_2020_063_01) and registered on the website ClinicalTrials.gov on 22/09/2021(NCT05053763). All patients who took part in the study provided written informed consent.

### Participants

We prospectively enrolled patients undergoing ERCP under non-intubation sedation from September 2021 to November 2021 at Nankai Hospital, Tianjin, China. The inclusion criteria were as follows: age range of 65–85 years; ASA physical status of grades I–III; BMI of 18-<30. The exclusion criteria were as follows: unable to provide consent; pregnancy; seriously abnormal renal function; previous report of abnormal surgical anesthesia recovery; consumption of antidepressant or monoamine oxidase inhibitor within 15 d; prolonged use of psychotropic substances or analgesics due to chronic pain; alcohol abusers, with known drug allergy, patients with anticipated difficult airway management, attended another medical trial within three months, or unable to communicate properly with the researchers.

### Intervention

The ERCP was performed with the patient placed at the left lateral decubitus position. During the procedure, supplemental oxygen (6 L/min) was administered to the patient by nasal cannula. Topical anesthesia was applied using 100 mg of dacronin spray, facilitating the endoscopy insertion before the sedation. At the same time, the heart rate (HR), electrocardiogram (ECG), end-tidal CO_2_, and peripheral oxygen saturation (SpO_2_) were constantly monitored. Also, every 5 min, noninvasive blood pressure (NIBP) was monitored automatically.

**Fig. 1:**
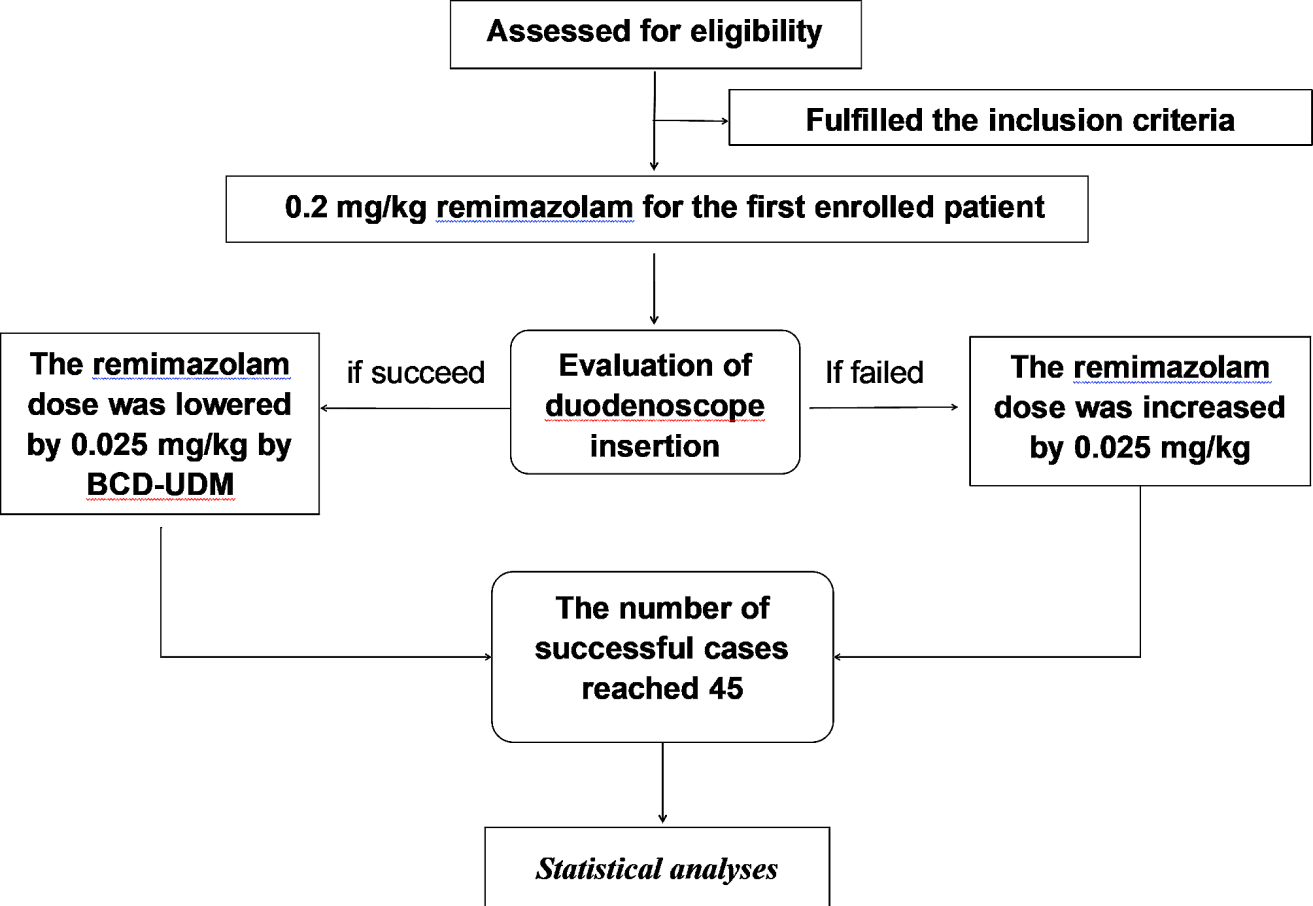
The flowchart of the participants in the study. BCD-UDM: Biased coin design up-and-down sequential method

An anesthesiologist and an anesthetic nurse were responsible for the patient during the whole procedure. According to our institute protocols,,alfentanil (dosage of 10 µg/kg; Yichang Renfu Pharmaceutical Co., Ltd.) was first administered for 3 min before administering sedative medication, and for definening the dose of remimazolam, we set the dose at 0.2 mg/kg for the first enrolled patient. Subsequently, in case the anestheisa induction was insufficient, we increased the dose of remimazolam by 0.025 mg/kg. If the sedation induction in a patient was successful, the following patient was randomized to either an identical dose (probability: 1-b = 0.89) or the dose was lowered by 0.025 mg/kg (probability: b = 0.11) according to the biased coin design up-and-down sequeential method (BCD-UDM) (Fig. 1).

The insertion of a duodenoscope was considered unsuccessful if the gross muscular movement was purposeful or when a patient coughed or vomited during the insertion. After 3 min of administering intravenous remimazolam, if modified observer’s assessment of alertness/sedation (MOAA/S) was > 1 and BIS was > 75, the induction was also considered unsuccessful and rescue sedation 1 mg/kg of propofol was administered. After the anestheisa induction, the sedation was maintained by continuous infusion of 0.2 to 1 mg/kg/h of remimazolam and 0 to 1 µg/kg/min of alfentanil to maintain the MOAA/S ranging between 1 and 2 [11]. 

During the procedure, hypotension was defined as MAP < 65 mmHg or a decrease of 20% from baseline, while hypertension was defined as MAP > 105 mmHg or an increase of 20% from baseline [11].Patients with hypotension and increased heart rate received a bolus dose of 50 µg phenylephrine, which was then titrated to BP and heart rate. In contrast, those with hypotension and HR < 60 bpm received a bolus dose of 10 mg ephedrine, which was then titrated to BP and heart rate. A bolus dose of 5 mg urapidil was administered and titrated to BP in hypertensive patients. If the HR was reduced to < 45 bpm, a 0.25 mg IV bolus dose of atropine was administered and titrated to HR. If it reached > 110 bpm, a bolus dose of esmolol (10 mg IV) was given and titrated to HR.

### Statistical analyses

For normally distributed data, continuous variables were presented as mean ± standard deviation (SD).The number of positive responses necessary for computing ED90 was at least 45. Thus, patients were prospectively enrolled until the number of successful insertions reached 45, and the number of opened envelopes (containing random dose assignments for successful insertions) totaled 44. A resident who did not participate in this study prepared the envelopes. Isotonic regression was used for estimating ED90, while the 95% CI was evaluated based on 2,000 bootstrap replicates. By using isotonic regression combined with bootstrapping CI, the data were further analyzed to determine the minimum effective dose necessary for successful sedation of 95% and 99% of the patients (ED95 and ED99). The dose estimator was used, which referred to the interpolated dose with an anticipated probability of effect equivalent to 0.9 in this study. The R 4.2.2 was used for performing statistical analyses.

## Results

**Fig. 2:**
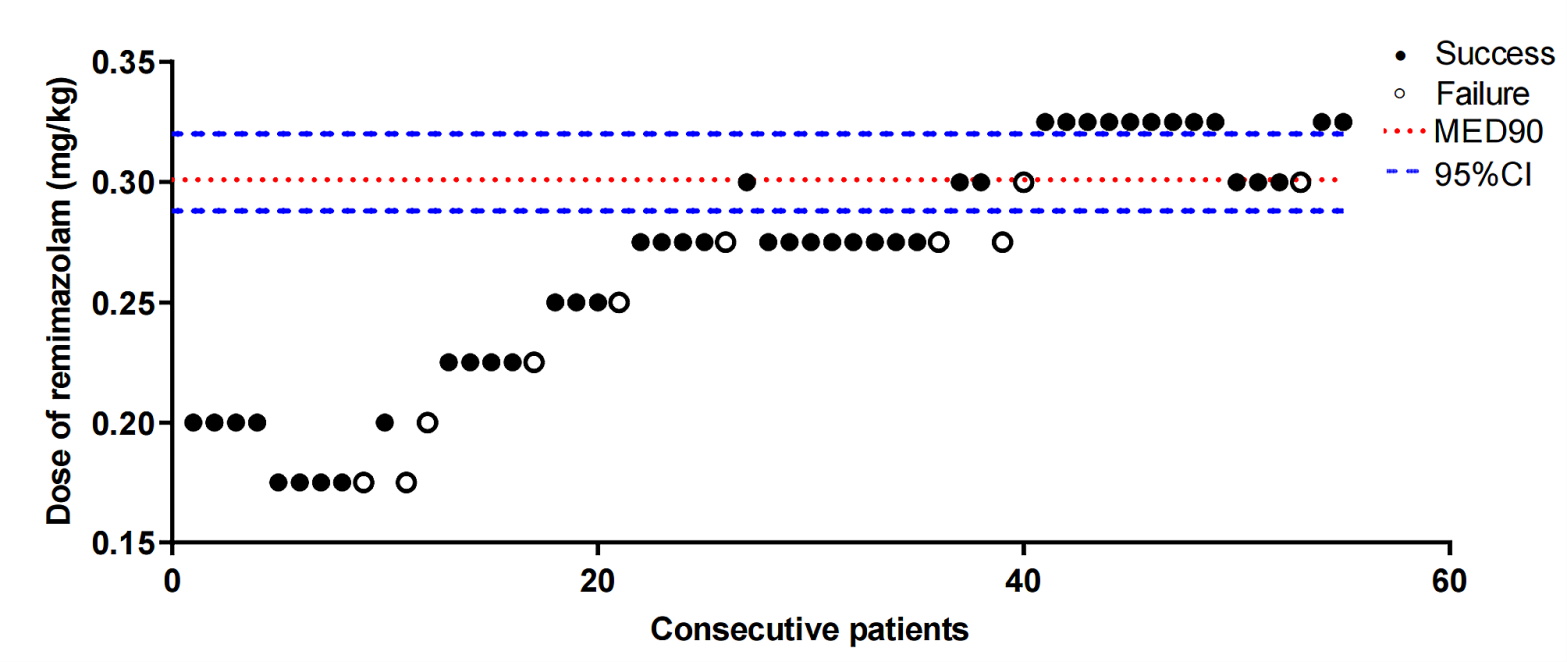
Graph of successful (solid spot) and failed (hollow spot) blocks with different dose of remimazolam. The horizontal line is the calculated effective dose of remimazolam providing successful insertion of a duodenoscope in 90% of patients during ERCP; error bars represent 95% CI

In total, 55 patients participated in this study (Table 1). Among them, the procedure failed in 10 patients during or after insertion of the duodenoscope, eight patients showed gross purposeful movement, and two patients experienced coughing. The up-and-down sequence with the biased coin design is shown in Fig. 2. ED90 was found to be 0.300 (95% CI :0.287–0.320). Further estimation showed that the ED95 was 0.315 (95% CI :0.312–0.323) and the ED99 was 0.323 (95% CI :0.323–0.325).

**Table 1:** Patients’characteristics

Variables	All(*n* = 55)	Success(*n* = 45)	Failure(*n* = 10)
Sex(M/F)	(23 / 32)	(22/23)	(1/9)
Age(years)	73.5 ± 5.3	73.7 ± 5.4	72.6 ± 4.9
ASA physicalstatus(I/II/III)	(4/42/9)	(3/34/8)	(1/8/1)
Weight(kg)	64.8 ± 11.0	66.2 ± 10.6	58.4 ± 10.6
Height(cm)	163.7 ± 7.9	164.4 ± 7.7	160.4 ± 8.5
BMI(kg/m2)	24.0 ± 2.6	24.4 ± 2.6	22.6 ± 2.3
BIS reach 75 time(s)	139.4 ± 12.3	139.3 ± 12.4	139.8 ± 12.2
Cause of failure			
Gross purposeful movement	-	-	8
Coughing	-	-	2

The response rates for various doses of remimazolam are presented in Table 2. The pooled-adjacent-violators algorithm (PAVA)-adjusted response rates were also determined. Using these rates, the monotonically non-decreasing response rates were generated for isotonic regression. The intra-anesthesia hemodynamic alterations during the successful insertion of a duodenoscope are presented in Table 3. Nine patients developed hypotension 10–15 min after injecting remimazolam, which was relieved by administering 5 mg of intravenous ephedrine. Two patients developed bradycardia, and one patient developed tachycardia. Hypoxia occurred in two patients among the 55 patients.

**Table 2:** Observed and pooled-adjacentviolators algorithm-adjusted response rates

Assigned dose	Successful insertion	Trials	Observed response rates	PAVA-adjusted response rates
0.175	4	6	0.667	0.667
0.200	5	6	0.833	0.789
0.225	4	5	0.800	0.789
0.250	3	4	0.750	0.789
0.275	12	15	0.800	0.789
0.300	6	8	0.750	0.789
0.325	11	11	1.0	1.0

PAVA, pooled-adjacent-violators algorithm.

**Table 3:** Hemodynamic changes during anesthesia induction in successful duodenoscopyinsertion(*n* = 45)

	T0	T1	T2	T3
MAP(mmHg)	99.0 ± 10.1	87.3 ± 9.8*	84.8 ± 10.7*	87.4 ± 9.6*
HR(beats/min)	76.1 ± 10.4	69.1 ± 8.3*	69.2 ± 8.0*	72.5 ± 10.5‡
SpO2(%)	99.9 ± 0.3	99.9 ± 0.3	99.9 ± 0.4	99.8 ± 0.6
BIS	96.9 ± 1.8	95.8 ± 2.4*	62.5 ± 3.8*†	65.9 ± 6.5*†‡

*P < 0.05 compared with T0,†*P* < 0.05 compared with T1, ‡*P* < 0.05 compared with T2.Values are means ± standard deviation. MAP, mean arterial blood pressure; HR, heart rate; SpO2,oxygen saturation; BIS, bispectral index; T0,base linevalue; T1,after remimazolam ministration; T2,immediately before duodenoscopy insertion; T3,5 min after duodenoscopy insertion.

For 45 successfully anesthetized patients, ERCP procedure was successfully completed without any additional sedative drugs during the induction phase of procedural sedation. Among the 10 patients who underwent unsuccessful duodenoscopy, most cases because of gross purposeful movement, ony two cases experiencing coughing, and 1 mg/kg of propofol was used as the additional sedative drug.

## Discussion

In this pilot study, we found that a loading dose of 0.300 mg/kg of remimazolam for elderly patients undergoing ERCP can safely, effectively, and quickly induce patients to fall asleep and inhibit responses to the insertion of a duodenoscope.

The ERCP was performed in patients by either sedation or general anesthesia [12, 13]. Due to the advantages of a shorter recovery time, better tolerance, and satisfaction among patients, anesthesiologists prefer the combination of propofol and opioids for sedation [14]. However, the major adverse consequence of propofol sedation is hypoxemia. Remimazolam showed a quicker onset and offset of the hypnotic effect, exhibiting cardiorespiratory stability [5]. Compared to midazolam, remimazolam had a better procedural sedation effect, achieved higher rates of success, and had a superior recovery profile. Most studies have emphasized the 50th quantile value of anesthetic remimazolam based on the Dixon-Mood up-and-down approach [15]. Clinically meaningful doses, however, are at higher quantiles, such as ED90, ED95, and ED99.

This is the first study to evaluate the ED90 of remimazolam for the insertion of a duodenoscope during ERCP. Given the poor precision of the Dixon-Mood approach at high quantiles, we used the biased coin up-and-down design for direct ED_90_ estimation. Next, isotonic regression and boot-strapping were performed to extrapolate ED90 to ED95 and ED99.

Although the administration of remimazolam-sufentanil is effective, surgical anesthesia is reliably administered, often by administering 0.1 µg/kg of sufentanil. In this study, 10 µg/kg of alfentanil was first administered, since a smaller dose was insufficient to precisely evaluate the effective dose [16]. Based on the PAVA-adjusted responses (Table 2), for patients aged 65–85 years, 0.3 mg/kg of remimazolam was suitable for effective sedation while ensuring safety, since, among our patients, the effectiveness of this dose was 75%. However, direct effective dose estimation is possible at any quantile with the biased coin design. Hence, direct estimation of ED95 and ED99 would be more accurate, although a large sample size might be required.None of the patients sedated with remimazolam experienced intraoperative awareness or painful injection during ERCP. Two patients developed oxygen desaturation, which is generally well-tolerated among patients with no underlying pulmonary problems.

Our results might not be universally applicable since the medications were administered by a single veteran anesthetist. Another shortcoming involves the definition of successful sedation. Insertion of the duodenoscope was considered unsuccessful when the gross muscular movement was purposeful or when a patient vomited or coughed during or after the insertion. When BIS was > 75 after 3 min of remimazolam injection, the procedure was considered to be a failure. Since the rate of success can vary based on the definition of successful sedation provided by the researchers, different studies should be compared with caution [17].

In conclusion, we found that the infusion of 10 µg/kg of alfentanil and 0.300 mg/kg of remimazolam can be performed for the sedation of 90% of aged patients undergoing ERCP for inhibiting responses to the insertion of a duodenoscope.

## Data Availability

The datasets used and/or analysed during the current study available from the corresponding author on reasonable request.

## References

[1] Cho CM (2021). The future of Endoscopic Retrograde Cholangiopancreatography in Korea. Gut Liver.

[2] Park CH, Park SW, Hyun B (2018). Efficacy and safety of etomidate-based sedation compared with propofol-based sedation during ERCP in low-risk patients: a double-blind, randomized, noninferiority trial. Gastrointest Endosc.

[3] Rex DK, Bhandari R, Desta T (2018). A phase III study evaluating the efficacy and safety of remimazolam (CNS 7056) compared with placebo and midazolam in patients undergoing colonoscopy. Gastrointest Endosc.

[4] Chen SH, Yuan TM, Zhang J (2021). Remimazolam tosilate in upper gastrointestinal endoscopy: a multicenter, randomized, non-inferiority, phase III trial. J Gastroenterol Hepatol.

[5] Lee A, Shirley M, Remimazolam (2021). A review in Procedural Sedation. Drugs.

[6] Sneyd JR, Rigby-Jones AE (2020). Remimazolam for anaesthesia or sedation. Curr Opin Anaesthesiol.

[7] Kim KM (2022). Remimazolam: pharmacological characteristics and clinical applications in anesthesiology. Anesth Pain Med (Seoul).

[8] Doi M, Morita K, Takeda J, Sakamoto A, Yamakage M, Suzuki T (2020). Efficacy and safety of remimazolam versus propofol for general anesthesia: a multicenter, single-blind, randomized, parallel-group, phase IIb/III trial. J Anesth.

[9] Pantos MM, Kennedy DR, Nemec EC. Remimazolam: a novel option for procedural sedation in high risk patients. J Pharm Pract. 2021:8971900211027303. 10.1177/08971900211027303 34155946

[10] White PF (2023). Remimazolam - can it become a cost-effective alternative to propofol for intravenous anesthesia and sedation?. J Clin Anesth.

[11] Dong SA, Guo Y, Liu SS, Wu LL, Wu LN, Song K, Wang JH, Chen HR, Li WZ, Li HX, Zhang L, Yu JB (2023). A randomized, controlled clinical trial comparing remimazolam to propofol when combined with alfentanil for sedation during ERCP procedures. J Clin Anesth.

[12] Smith ZL, Das KK, Kushnir VM (2019). Anesthesia-administered sedation for endoscopic retrograde cholangiopancreatography: monitored anesthesia care or general endotracheal anesthesia?. Curr Opin Anaesthesiol.

[13] Dhaliwal A, Dhindsa BS, Saghir SM (2021). Choice of sedation in endoscopic retrograde cholangiopancreatography: is monitored anesthesia care as safe as general anesthesia? A systematic review and meta-analysis. Ann Gastroenterol.

[14] Cheriyan DG, Byrne MF (2014). Propofol use in endoscopic retrograde cholangiopancreatography and endoscopic ultrasound. World J Gastroenterol.

[15] Liu M, Sun Y, Zhou L, Feng K, Wang T, Feng X (2022). The median effective dose and Bispectral Index of Remimazolam Tosilate for Anesthesia induction in Elderly patients: an Up-and-down sequential allocation trial. Clin Interv Aging.

[16] Yu AL, Critchley LA, Lee A, Gin T (2006). Alfentanil dosage when inserting the classic laryngeal mask airway. Anesthesiology.

[17] George RB, McKeen D, Columb MO, Habib AS (2010). Up-down determination of the 90% effective dose of phenylephrine for the treatment of spinal anesthesia-induced hypotension in parturients undergoing cesarean delivery. Anesth Analg.

